# A high-throughput technique to map cell images to cell positions using a 3D imaging flow cytometer

**DOI:** 10.1073/pnas.2118068119

**Published:** 2022-02-16

**Authors:** Zunming Zhang, Rui Tang, Xinyu Chen, Lauren Waller, Alston Kau, Anthony A. Fung, Bien Gutierrez, Cheolhong An, Sung Hwan Cho, Lingyan Shi, Yu-Hwa Lo

**Affiliations:** ^a^Department of Electrical and Computer Engineering, University of California San Diego, La Jolla, CA 92093;; ^b^Department of Bioengineering, University of California San Diego, La Jolla, CA 92093;; ^c^NanoCellect Biomedical Inc., San Diego, CA 92121

**Keywords:** single cell, high throughput, 3D imaging flow cytometer, disease diagnosis, spatial biology

## Abstract

We develop a high-throughput technique to relate positions of individual cells to their three-dimensional (3D) imaging features with single-cell resolution. The technique is particularly suitable for nonadherent cells where existing spatial biology methodologies relating cell properties to their positions in a solid tissue do not apply. Our design consists of two parts, as follows: recording 3D cell images at high throughput (500 to 1,000 cells/s) using a custom 3D imaging flow cytometer (3D-IFC) and dispensing cells in a first-in–first-out (FIFO) manner using a robotic cell placement platform (CPP). To prevent errors due to violations of the FIFO principle, we invented a method that uses marker beads and DNA sequencing software to detect errors. Experiments with human cancer cell lines demonstrate the feasibility of mapping 3D side scattering and fluorescent images, as well as two-dimensional (2D) transmission images of cells to their locations on the membrane filter for around 100,000 cells in less than 10 min. While the current work uses our specially designed 3D imaging flow cytometer to produce 3D cell images, our methodology can support other imaging modalities. The technology and method form a bridge between single-cell image analysis and single-cell molecular analysis.

## Significance

This article demonstrates a high-throughput technique to map cell images to cell positions. The technology uses a three-dimensional (3D) imaging flow cytometer to record multiparameter 3D cell images at a throughput of 1,000 cells/s and a cell placement robot to place the exiting cells from the imaging system on a filter plate in a first-in–first-out manner so the cells on the plate have the same order as the cells that are imaged. Innovative algorithms were developed to match the cell sequences from the imaging and placement modules to detect and eliminate errors to ensure high accuracy. The technology forms an unprecedented bridge between single-cell molecular analysis and single-cell image analysis to connect phenotype and genotype analysis with single-cell resolution.

The isolation and analysis of single cells from a heterogeneous cell population have impacted biomedical research profoundly (1, 2). Single-cell analysis can be broadly divided into two areas, namely, single-cell genomics and single-cell high-content microscopy (3–5). The former deciphers the genomic and phenotypical information by detecting gene expressions, mutations, and genetic aberrations in individual cells (6–9). The latter provides high-resolution spatial and morphological information and cell-cell interactions (10). While the toolsets for both approaches have advanced significantly, one remaining technology gap is the lack of effective tools that can connect the two types of single-cell information. That is, no effective tool directly relates the morphological properties to the genomic properties of the very same cell. The emerging field of spatial biology aims to solve this issue via DNA barcoding technologies (11–15). However, current methods such as the 10× Visium platform are unable to resolve single cells (16, 17). Microlaser dissectors coupled with a high-resolution microscope can provide single-cell resolution, but the throughput is much too slow to be incorporated into the single-cell workflow in most practical applications (18–20). Above all, all current spatial biology techniques cannot address the problem for nonadherent cells such as lymphocytes, for which the connection between the phenotype and morphology and immune response of the same T cell can be particularly insightful.

The recent advances in the image-guided cell sorter or image-guided fluorescence-activated cell sorting (FACS) system have made good strides toward this goal (21–24). By isolating cells of the same image features based on a predefined gating criterion, one can perform a downstream genomic analysis with cells of similar imaging characteristics (21, 22, 24, 25). However, today’s image-guided FACS produces only two-dimensional (2D) images, lacking the high information contents of three-dimensional (3D) imaging modalities such as confocal microscopes and light-sheet microscopes. Few imaging flow cytometers can produce high-content 3D images of single cells (26–29), and none of them, to our knowledge, is able to isolate cells based on the 3D image features due to the technological incompatibility between 3D imagining optics, cell sorting devices, and the great challenges in real-time processing of 3D images required for 3D image-guided cell sorting.

To address the above technology gap, we propose and demonstrate an approach that bypasses the requirements for real-time 3D image processing and cell sorting. Our approach combines two key hardware components, namely, a 3D imaging flow cytometer (3D-IFC) and a cell placement robot.

We introduced suspended cells into a 3D-IFC to record multiparameter 3D cell images at a throughput as high as 1,000 cells/s. The cells exiting the 3D-IFC system were directed to a cell dispensing robot that placed the exiting cells onto a transparent filter plate in such manners that the cells placed on the filter plate follow the same order in which the cells were imaged by the 3D-IFC. In other words, there is a one-to-one correspondence between the recorded 3D cell image by the 3D-IFC and the position of the cell on the filter plate. However, we need to overcome the follows two obstacles to realize this concept: 1) matching the sequence of hundreds of thousands of cell images from the 3D-IFC to the sequence of (ideally) the same number of cells deposited on the plate and 2) detecting any errors between the two long sequences to prevent error propagation and accumulation.

To address the first challenge, we introduced three types of marker beads of distinctive features that can be recognized by an off-the-shelf imager on the cell placement robot to match their sequence to the readout from the 3D-IFC. By assigning each marker bead a symbol (A, T, C) used for DNA sequencing, we were able to use the existing bioinformatics toolbox to sequence and match the two long sequences from the 3D-IFC and the cell plate. These marker beads serve analogously to introns, and the cells of interest between the marker beads can be regarded as exons. Essentially, we used the marker bead sequence (i.e., introns) to align the two long sequences and then interrogate the regions between the marker beads to analyze the cells based on their 3D images. In this approach, we can fully leverage the established bioinformatics tools to support data streams of essentially any length. To address the second challenge, we have developed a robust and efficient error detection methodology to identify two major types of errors—deletion errors and misplacement errors—that can occur in our operation scenario. The detailed algorithm and its results are presented in *Results*.

Overall, we have developed an approach to bridge the technology gap of relating single-cell molecular analyses to single-cell imaging of nonadherent cells in which imaged cells are available for not only genomic analysis but also other applications as well, including the formation of single cell-derived microcolonies, drug response studies, and metabolic and cell secretion analyses. It is worth mentioning that although we used 3D-IFC as a high-throughput imaging tool to acquire cell images here, the methodology is general and can be readily applied to other imaging devices such as 2D imaging cytometers and optical microscopes that can capture images of moving objects and be interfaced with a dispensing system following the first-in–first-out (FIFO) rule in general. Compared to image-guided FACS systems, our design can keep all cells entering the system on a cell-friendly plate to support various downstream analyses and cell processing and allow researchers to retrieve any cells in the system at different times and for different purposes. Finally, we take the approach of cell imaging before placement instead of cell placement before imaging because the former is compatible with the high-throughput flow cytometers as the mainstay of nonadherent single-cell analysis. Our work demonstrates a workflow and technology that enrich the field of single-cell research and spatial biology.

## Results

### Aligning Bead/Cell Sequences to Map Cell Images to Their Positions.

**Fig. 1.**
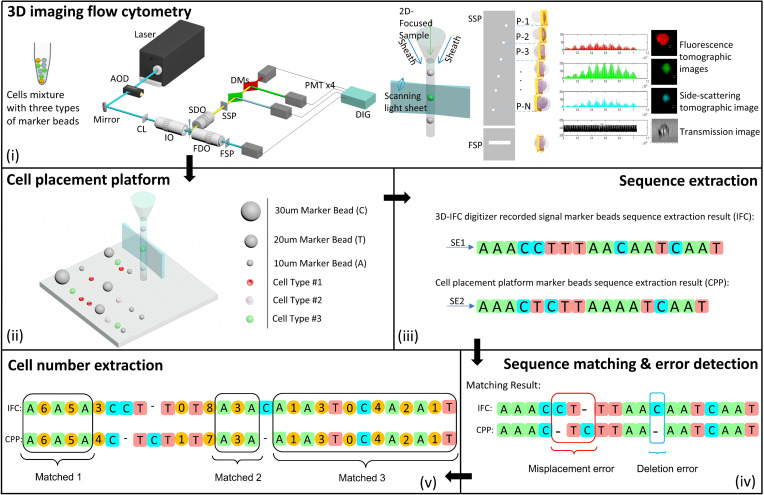
Single-cell analysis and isolation workflow based on 3D-IFC and robotic cell dispensing and pickup. The workflow is as follows. (*i*) Three types of marker beads are mixed with a cell mixture to help map cell images from the image stream to cell locations. The sample is run through a 3D-IFC that can capture 3D fluorescent and SSC images as well as 2D cell transmission images. (*ii*) After cells exit the 3D-IFC, they are dispensed by a robotic dispenser in a FIFO manner on a template. (*iii*) Marker beads sequences from the 3D-IFC signal and the CPP are extracted using developed sequence extraction pipelines described in *SI Appendix*, Figs. S1 and S2. (*iv*) Two sequences are compared and matched using a sequencing bioinformatics tool. (*v*) Marker beads sequence errors (including deletion and misplacement errors) are detected, and for the correctly registered marker beads, the number of cells between two marker beads is identified. In this manner, one can relate hundreds of thousands of individual cells to their respective 3D fluorescent and scattering images in high accuracy. If certain cells with specific image features are of interest, one can locate and pick up those cells individually for downstream analyses. AOD, acousto-optic deflector; CL, cylindrical lens; IO, 20×/0.42 illumination objective; SDO, 10×/0.28 side detection objective; SSP, side spatial filter; DMs, dichroic mirrors; FDO, forward detection objective; FSP, forward spatial filter; DIG,125 MSs−1 digitizer; PMT, photomultiplier tube; P-1, P-2, P-3, P-N, pinhole positions; SE1, sequence extraction from 3D-IFC, SE2, sequence extraction from CPP.

Fig. 1 shows the overall design and workflow of our approach. The system consists of two interconnected hardware modules, namely, a 3D-IFC and a robotic cell dispenser. Cells and beads were premixed and examined using the 3D-IFC system. The 3D hydrodynamically focused sample flow establishes a single-cell stream with a sample concentration of ∼500 samples/µL. When a cell or bead passes through the laser interrogation area, it is illuminated by a scanning light-sheet at a 200-kHz scanning rate. The spatial filter placed at the image plane contains a series of spatially positioned pinholes aligned with the cell flow direction by a predetermined separation. The emitted light from a specific portion of a cell is detected by photomultiplier tubes (PMTs). The spatial-temporal transformation is applied to reconstruct the 3D tomographic images. The forward spatial filter contains a long slit aligned with the laser scanning range. The transmitted light is collected by a PMT, and the signal can produce a 2D transmission image. In this cameraless design with a scanning light-sheet and spatial masks, the 3D-IFC system can produce 3D side scattering (SSC) and fluorescent images plus a 2D transmission image of traveling cells at a rate of 1,000 cells/s. The details of the 3D-IFC can be found in our earlier publications (29, 30).

The robotic cell placement platform (CPP) contains a three-axis motorized stage and a holder. The moving speed of the motorized stage is programmable to control the cell-to-cell spacing and can be up to 75 mm/s. The cells exiting the 3D-IFC reside on a transparent porous film on the sample holder that has an array of groves connected to a vacuum pump. The liquid out of the 3D-IFC is immediately absorbed by the porous membrane filter through the capillary effect, and the extra liquid is drained by vacuuming the groves under the membrane.

The cell sample was premixed with three nonfluorescent beads of different sizes, as follows: 10-µm beads which we represented as nucleobase A, 20-µm beads which we represented as nucleobase T, and 30-µm beads which we represented as nucleobase C. Hence, the sequence consists of these three types of marker beads and cells. By matching the marker bead sequences between the 3D-IFC signals and CPP, we were able to align the two sequences, which subsequently enabled us to map the cells between marker beads. To keep the average number of cells between marker beads to be a relatively small number ($\bar{n}=2$) and minimize the chance of error, we kept the ratio between cells and the total number of marker beads to be 2:1.

When the cells and beads passed the laser interrogation area of the 3D-IFC and exited the flow cell, they were dispensed in a FIFO manner on a template consisting of a 12-µm-thick transparent porous film (Sterlitech, Stock Keeping Unit 1300026) on a holder with an array of groves. The liquid out of the 3D-IFC (at around 300 µL/min) was immediately absorbed by the porous membrane filter through the capillary effect and drained by vacuuming the groves under the membrane. Only the beads and cells were left on the wetted porous membrane. The moving speed of the template was programmed according to the cell density in the sample to achieve an average cell-to-cell spacing of 250 µm along the line of travel and a spacing of 500 µm between two adjacent lines of cells. In this design, a filter plate of the same size as a 384-well plate can house around 6 × 10^4^ cells. For a 30-min run of the 3D-IFC at a throughput of 300 cells/s, we can record around 500,000 3D images of single cells deposited on 10 cell plates with full knowledge of the position of every single cell and its 3D image.

**Fig. 2.**
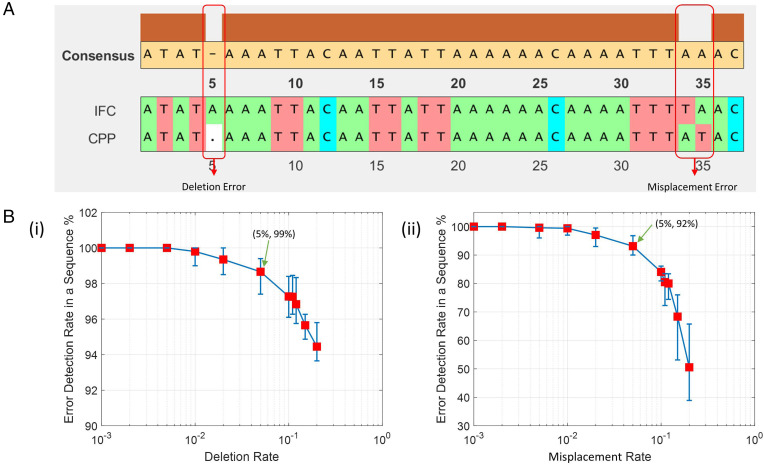
(*A*) Example sequence matching results of the marker bead readout by the 3D-IFC and the CPP using the bioinformatics toolbox in MATLAB. (*B*) Monte Carlo–simulated error detection probability versus error rate due to (*i*) deletion (a marker bead is missing) and (*ii*) misplacement (a marker bead is misplaced). Here, 10-µm marker beads are represented by A, 20-µm marker beads by T, and 30-µm marker beads by C.

We used the bioinformatics toolbox to match the bead sequences of the 3D-IFC readout and the cell plate, which is equivalent to comparing two DNA sequences. We first matched the marker beads between the two sequences and then matched the cell numbers between marker beads. An example of the matched result of the marker beads is shown in Fig. 2*A*. The consensus map in Fig. 2 shows that errors can be detected and located. Errors can be caused by bead/cell trapping within the system (equivalent to deletion error) or misplacement (i.e., violating the FIFO rule). In this example, we found one deletion error (in the 5th position) and one misplacement error (in the 34th position) in Fig. 2*A*.

If errors are found in the marker bead sequences, we will skip the cells following the erroneous marker beads. If errors occur to the cells between two marker beads, we will disregard the cells between the marker beads to assure high accuracy. In this approach, we minimize the probability of assigning wrong images to the cells. In a typical run, we were able to relate cell images to >80% of the cells with high confidence, dropping about 15 to 20% of cells due to deletion or misplacement errors. Next, we present simulation results of how the bioinformatics software can detect deletion and misplacement errors and how the error detection capability changes with the frequency of these errors.

Fig. 2 *B*, *i* and *ii* show Monte Carlo simulations of the dependence of error detection capability on the error occurring frequency for both deletion errors and misplacement errors. In the simulations, we assumed the first DNA sequence contained three types of nucleotides for a total length of 10,000 nucleotides. The second DNA sequence was generated using the first one as the template, but some nucleotides were deleted or misplaced at given error rates (e.g., randomly deleted 100 from 10,000 nucleotides for a 1% deletion rate). Two sequences of DNAs were then matched using the bioinformatics toolbox. The simulations showed that for a 5% probability of deletion error, 99% of the errors were successfully located. For a 5% probability of misplacement error, 92% of the errors were successfully located. If the probability of both types of errors was within 1% (i.e., less than 100 deletion or misplacement errors in a sequence of 10,000 nucleotides), all errors were detected. For those regions of high deletion and misplacement errors that led to lower than a threshold confidence level, all cells and their images in those regions would be disregarded.

### Experiment with a Mixture of Marker Beads and Fluorescent Signal Beads.

**Fig. 3.**
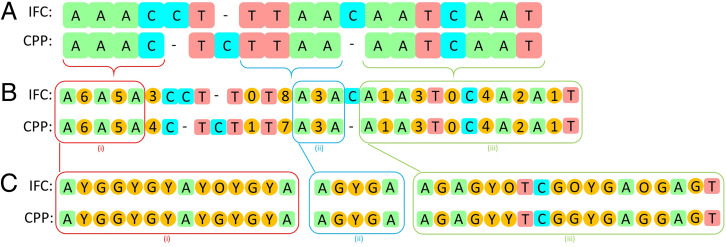
Proof-of-concept experiment with fluorescent beads (Y, Envy Green; G, Dragon Green) mixed with marker beads (A, T, C). (*A*) Matching results of the marker bead sequences from the 3D-IFC readout and CPP readout using the bioinformatics toolbox in MATLAB. (*B*) Map including the number of fluorescent beads between correctly identified marker beads. (*C*) Detailed readout of the fluorescent beads (Y, G) between marker beads (A, T, C). In *C*, besides the Y and G, “O” represents beads of which the 3D-IFC images are out of focus. In other words, even if the bead can be identified, its image is too blurred to extract useful information. (*i*), (*ii*), and (*iii*) indicate sections in *B* that are expanded in *C*.

For an initial feasibility study of matching images with their positions, we mixed two types of fluorescent polystyrene beads (10-μm Dragon Green and 10-μm Envy Green from Bangs Laboratories) with the marker beads in a 2:1 ratio. After extracting the two sequences from the 3D-IFC readout and the CPP readout, we used the bioinformatics toolbox in MATLAB to match the two sequences. A section of the matched sequences is shown in Fig. 3*A*. In this section, we found one misplacement error (in the 5th position) and one deletion error (in the 12th position) in Fig. 3*A*.

Fig. 3*B* lists the number of fluorescent beads between each pair of marker beads, and Fig. 3*C* shows the actual readouts of the fluorescent beads to be Envy Green (Y) or Dragon Green (G). In practical operations, we needed to introduce another symbol “O” for those objects for which the images were out of focus. Due to an imperfect flow confinement in our 3D-IFC system, occasionally some objects were deviated from the center of the cuvette to produce out-of-focus images. Combining all these factors, including deletion and misplacement of marker beads and fluorescent beads and out-of-focus images, we were able to accurately locate 80% of the cells with their 3D images.

### Tracking 3D Cell Images and Their Positions.

Next, we demonstrate the ability to map 3D images of labeled and unlabeled cells to their positions. The test sample contained a mixture of human embryonic kidney 293 (HEK-293), Michigan Cancer Foundation-7 (MCF-7), and cervical cancer (HeLa) cells in an ∼1:1:1 ratio. MCF-7 cells and HeLa cells were fluorescently stained with the carboxyfluorescein succinimidyl ester (CFSE) (excitation/emission [Ex/Em] wavelengths: 492/517 nm, Thermo Fisher) and the CellTrace yellow proliferation kit (Ex/Em 546/579 nm, Thermo Fisher), respectively. The HEK-293 cells were unstained.

**Fig. 4.**
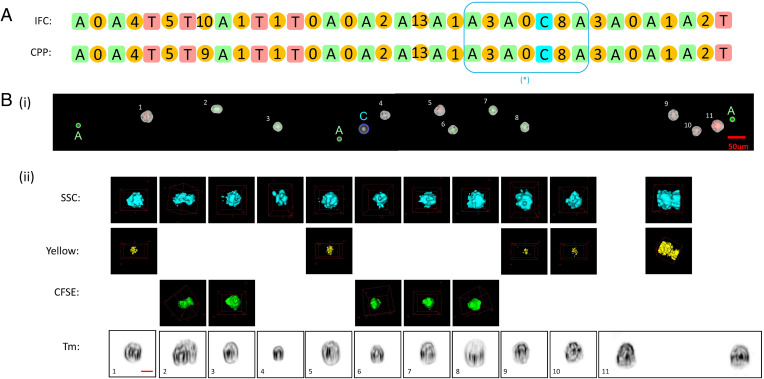
Human cancer cell experiment. MCF-7 and HeLa cells were stained with CFSE and CellTrace yellow, respectively, and HEK-293 were unstained. (*A*) Representative section of the matched sequences from the IFC and CPP readouts. The number between the adjacent marker beads shows the number of cells in between. When the number of cells in two sequences matches, we can unambiguously match the cell with its 3D images. [* indicates section that is expanded in *B* (*i*) and (*ii*)]. (*B*) A representative section (in the box in *A*) of cells (*i*) on the CPP membrane and (*ii*) having their 3D SSC images and fluorescent images (in yellow and green from CellTrace Yellow and CFSE, respectively) as well as 2D transmission images. Notice that cell 2 appears to be a doublet. Cell 11 shows a doublet in the scattering and fluorescent images, but the 2D transmission image can resolve the doublet from the perspective. CFSE, Cell Proliferation Kit (Ex/Em, 488/517); yellow, CellTrace yellow proliferation kit (Ex/Em, 546/579); SSC, side scattering (90 degrees); Tm, transmission image. (Scale bar, 10 µm.)

Flowing the 3D-IFC system for high-throughput imaging, cells were dispensed onto the membrane filter by the robotic system in a FIFO manner. We first checked the sequence of marker beads using the bioinformatics toolbox to identify all matched pairs and excluded those mismatched marker beads due to deletion or misplacement errors. Next, between two adjacent marker beads, we compared the number of cells from the 3D-IFC images and the cell plate and marked those sections where the two cell numbers are matched, dropping the sections where the cell numbers are not matched. Fig. 4*A* shows the number of cells between two adjacent marker beads, and Fig. 4*B* locates the cells on the plate and their 3D SSC fluorescent images and 2D transmission images. The built-in camera in the robotic cell placement module has a large field of view but low resolution, as its purpose is to simply identify the positions of marker beads and cells. Aided by image processing algorithms, the low-resolution camera images could distinguish marker beads by their size and registered cells and their location coordinates, which were later used for targeted cell pickups. The image processing algorithm was also able to distinguish beads and cells from the background patterns of the pores on the filter surface.

With the cell mapping capabilities, we can record the 3D image of each cell and locate it on the cell placement platform with its position coordinates for retrieval and molecular analysis or cell-based assay. Notably, in Fig. 4, cell 2 appears to be a doublet, as shown from its SSC and CFSE fluorescent images as well as 2D transmission images. However, cell 11 shows a doublet in its SSC and yellow fluorescent images but not in its 2D transmission image from the perspective. This example shows that 2D images could run into the issues of occlusion and perspective compared with 3D tomography.

### Separating Breast Cancer Cells from Normal Cells.

**Fig. 5.**
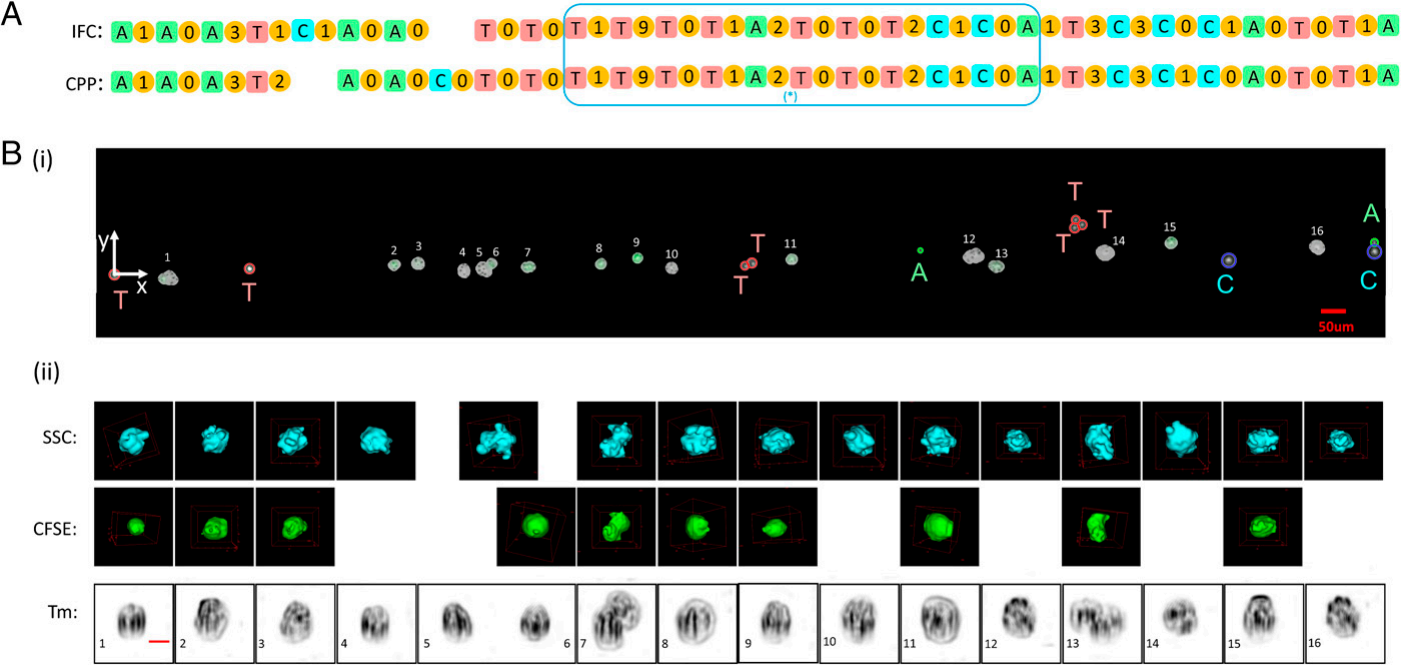
Breast cancer cell experiment. (*A*) Representative section of the matched sequences from the IFC and CPP readouts. The number between adjacent marker beads shows the number of cells in between. When the number of cells in two sequences matches, we can unambiguously match the cell with its 3D images. (*B*) Details of marker bead and cell images within the highlighted box in *A*. (*i*) Low-resolution camera images for marker beads and cells on the membrane filter of CPP. (*ii*) High-resolution 3D SSC and fluorescent images and 2D transmission images of MCF-7 (CFSE stained) and MCF-10A (unstained) cells from the 3D-IFC. The 3D-IFC images can detect cell doublets (cell 7 and cell 13). For cell 5, the doublet consists of an MCF-7 (fluorescent) and an MCF-10A (nonfluorescent) cell. For cell 13, the doublet consists of two MCF-7 (fluorescent) cells. (Scale bar, 10 µm.)

Here, we identify and locate human breast cancer cells (MCF-7) from human breast epithelial cells (MCF-10A) by mapping cell locations to their images. The MCF-7 cells were fluorescently stained with the CFSE cell proliferation kit, and MCF-10A cells were not stained. We fluorescently labeled MCF-7 cells to establish the ground truth for verification. After capturing the 3D image of each cell by the 3D-IFC at a rate of 300 cells/s, cells were dispensed on the membrane in a FIFO manner. Fig. 5*A* shows the sequence of marker beads and the cell numbers between two marker beads. Good matches between the 3D-IFC and the cell plate were obtained in most regions except a single misplacement of the marker bead C. The cells on the membrane and their corresponding 3D images in the matched regions are shown in Fig. 5*B*. Note that the 3D-IFC images are also able to detect cell doublets (e.g., cell images 7 and 13 in Fig. 5). This can find important applications in identifying a T cell/cancer cell complex for T cell receptor and neoantigen detection, a key step for Chimeric antigen receptor (CAR)T immunotherapy.

### Liver Disease Detection.

Liver disease is a global healthcare burden, causing millions of deaths per year worldwide. The progression of liver disease could be divided into several stages (31). The hepatitis stellate cells (SCs) from patients with early-stage liver disease would be a mixture of normal liver cells and cells in different stages of liver disease (32). Early-stage liver disease analysis and isolation are not only important in managing the disease but also beneficial in liver drug discovery and personalized medicine. Our technique offers the capability of imaging and isolating individual cells, which enable disease diagnosis and applications in drug discovery.

In a proof-of-concept experiment, the biopsy-proven nonalcoholic steatohepatitis (NASH) SC derived from a patient with early fibrosis stage 1/2 and SC from a healthy control were studied. We first ran both NASH SC and control SC separately to collect cell images. A total of 11 morphological features from both 2D transmission images and 3D SSC images were extracted from these cell images by offline analysis. We applied the unsupervised *k*-means clustering algorithm using the features from NASH SC to separate normal liver cells from cells with liver disease. Then, the features from control SC were used to examine the model clustering performance.

**Fig. 6.**
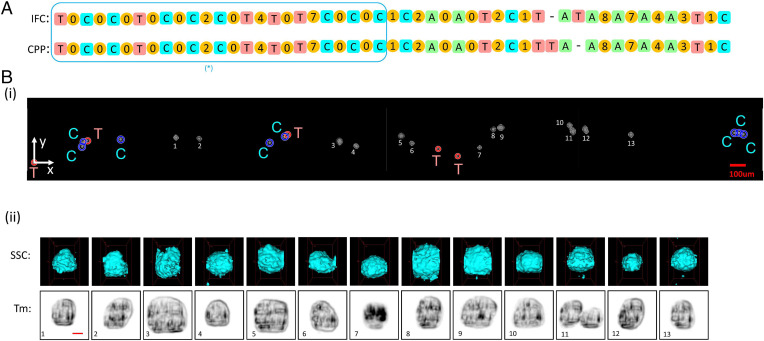
Liver cell experiment. (*A*) Representative section of the matched sequences from the IFC and CPP readouts. The number between adjacent marker beads shows the number of cells in between. When the number of cells in two sequences matches, we can unambiguously match the cell with its 3D images. (*B*) Details of marker bead and cell images within the highlighted box in *A*. (*i*) Low-resolution camera images for marker beads and cells on the membrane filter of CPP. The image resolution is sufficient for the identification of the marker beads and the coordinates of the cells as indicated in Table 1. (*ii*) High-resolution 3D SSC and 2D transmission images of liver cells from the 3D-IFC. The images can detect cell doublets (cell 11). (Scale bar, 10 µm.)

**Fig. 7.**
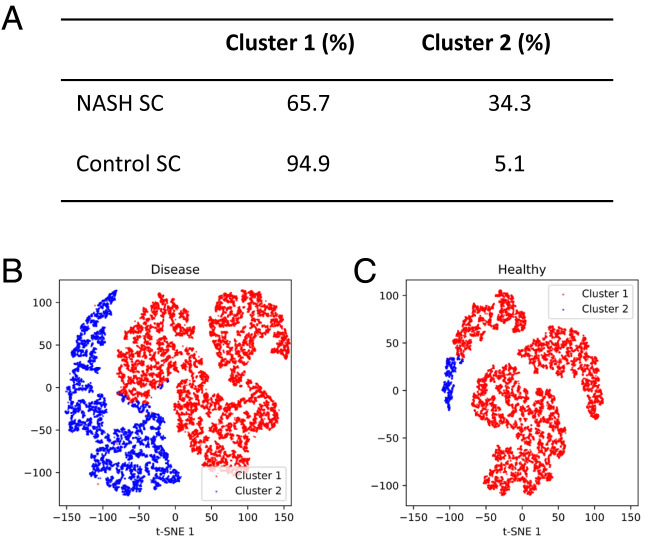
Liver cell clustering from healthy and early-stage NASH samples. (*A*) Cluster distribution of the NASH SC and the control SC. The clustering model was fitted using the dataset from the NASH HC. Then, the model was used to predict the dataset from the control SC. (*B*) t-SNE visualization of the clustering result of the NASH SC dataset. (*C*) t-SNE visualization of the clustering result of the control SC dataset. Both datasets contain ∼15,000 3D cell images. HC, healthy control; SC, stellate cell; t-SNE, t-distributed stochastic neighbor embedding.

We demonstrated the system capability to identify and locate each cell after capturing the 2D and 3D cell images by the 3D-IFC at a rate of 300 cells/s. The NASH SC sample was mixed with marker beads, and cells were dispensed on the membrane in a FIFO manner. Fig. 6*A* shows the sequence of marker beads and the cell numbers between two marker beads. The cells on the membrane and their corresponding 3D images in the matched regions are shown in Fig. 6*B*. Note that cell 11 is a doublet. Fig. 7 shows the clustering results and the corresponding t-SNE visualizations for NASH SC and control SC.

To pick up any selected cells based on their image features, we need to locate the cell positions, which are random on the porous membrane due to several factors, including the Poisson statistics of the time when cells exit the 3D-IFC system, the finite size of the dispensing tip, and the relatively large amount of sheath flow that carries the cells. The ability of an automatic generation of cell position coordinates makes selective cell pickup fast and easy. To measure the location of the marker beads and cells on the membrane, we have developed image processing algorithms to identify the marker beads by size and record the coordinates of each marker bead and cells. The software is capable of distinguishing beads and cells from background features of the porous membrane. *SI Appendix* shows how those background patterns of the porous membrane can be removed to produce images in Figs. 4*B*, 5*B*, and 6*B*.

As an example of using the low-resolution, wide field-of-view imager in the CPP to register the position of the marker beads and cells, Table 1 lists the coordinates of the marker beads and cells on the membrane filter in Fig. 6*B*. The imager has sufficient resolution to resolve the marker beads and the presence of cells between the marker beads and to register their positions with an accuracy of ±10 μm, which is sufficient for the cell pickup tools. Table 1 also lists the extracted features and the clustering result. One can pick up any chosen cells, for example, cells belong to cluster 2 on the membrane according to their coordinates, and transfer them to a common platform such as a 384-well plate for downstream analyses. There are many automated cell pickup heads to pick up single cells by aspiration with a high rate and efficiency (33, 34). One can also introduce different treatments to certain cells based on their image analysis results and locations, which would be beneficial in the drug discovery area.

**Table 1.** Registration of marker beads and cells in the liver cell experiment (Fig. 5) by their position coordinate on the membrane filter and their corresponding feature extraction and clustering results

Cell or marker bead	Position (x, y), µm	Transmission image (2D), µm				SSC image (3D)		Clustering result
		Surface area	Major axis	Minor axis	Perimeter	Volume, µm^3^	Surface area, µm^2^	
Marker bead #1 (T)	(0, 0)	—	—	—	—	—	—	
Marker bead #2 (C)	(299, 98)	—	—	—	—	—	—	
Marker bead #3 (C)	(307, 130)	—	—	—	—	—	—	
Marker bead #4 (T)	(334, 135)	—	—	—	—	—	—	
Marker bead #5 (C)	(541, 143)	—	—	—	—	—	—	
Cell #1	(887, 155)	464.5	26.4	22.7	76.9	6,495	1,604	1
Cell #2	(1,031, 149)	648.5	33.2	24.9	92.3	10,367	4,532	1
Marker bead #6 (C)	(1,480, 120)	—	—	—	—	—	—	
Marker bead #7 (C)	(1,576, 160)	—	—	—	—	—	—	
Marker bead #8 (T)	(1,593, 172)	—	—	—	—	—	—	
Cell #3	(1,920, 130)	1,125.3	39.2	36.8	122.1	23,150	7,705	2
Cell #4	(2,026, 100)	506.0	26.8	24.3	80.0	7,160	2,359	1
Cell #5	(2,313, 163)	909.7	34.4	34.2	109.3	19,712	4,842	1
Cell #6	(2,379, 118)	645.8	31.9	26.0	90.6	10,422	3,994	1
Marker bead #9 (T)	(2,543, 83)	—	—	—	—	—	—	
Marker bead #10 (T)	(2,666, 37)	—	—	—	—	—	—	
Cell #7	(2,804, 91)	394.0	23.8	21.7	74.8	5,327	2,471	1
Cell #8	(2,890, 206)	586.5	32.2	23.4	90.5	8,442	3,907	1
Cell #9	(2,937, 217)	814.5	35.0	29.8	103.7	15,181	3,705	1
Cell #10	(3,368, 232)	683.5	30.9	28.7	96.6	12,398	3,876	1
Cell #11	(3,390, 190)	—	—	—	—	—	—	—
Cell #12	(3,475, 194)	596.8	31.8	24.0	87.9	8,791	3,075	1
Cell #13	(3,753, 177)	506.0	28.0	23.2	81.1	7,210	2,396	1
Marker bead #1 (C)	(4,402, 187)	—	—	—	—	—	—	
Marker bead #1 (C)	(4,431, 187)	—	—	—	—	—	—	
Marker bead #1 (C)	(4,459, 178)	—	—	—	—	—	—	

—, not of interest.

## Discussion

This technique relates 3D imaging features of individual nonadherent cells at high throughput to their spatial coordinates on a plate. Our design consists of two parts, as follows: recording 3D cell images at high throughput (up to 1,000 cells/s) using a 3D-IFC and dispensing cells in a FIFO manner using a robotic CPP. When hundreds of thousands of cells pass the system in continuous operation for 10 min, errors due to violations of the FIFO principle are inevitable due to occasional cell trapping inside the system or cell order scrambling due to disruption of the laminar flow. To prevent the incorrect mapping of cell images to their positions on the cell placement membrane, we invented a method that used marker beads and DNA sequencing software to detect errors and discard any portions with high error probabilities. Using this method, one can detect any errors and isolate the erroneous regions to prevent error propagation and accumulation. Proof-of-concept experiments with human cancer cell lines and healthy/diseased liver cells were performed to demonstrate the feasibility of the approach. Over 100,000 cells placed on the cell plates can be located based on their 3D SSC and fluorescent images, as well as 2D transmission images. Since the position coordinate of every single cell on the cell placement membrane is recorded, our technique also allows users to pick up cells of specific phenotypes for cell-based assays, culturing, and downstream molecular analyses such as RNA sequencing, proteomic, and metabolic analyses. Given that several commercial devices are already available for efficient cell pickup and dispensing into 384-well plates, our paper does not cover any specific cell pickup methods but shows the highly efficient approach to generate cell position coordinates. In our design, cells are placed on a porous membrane to keep cells wetted by the culture medium and easily aspirated without rupturing the cell membrane or excessive stress. Finally, while the current work uses our in-house-designed 3D-IFC to produce the cell image data, our methodology is general enough to support other imaging modalities such as the commercial 2D imaging flow cytometer (e.g., Amnis ImageStream system).

## Materials and Methods

### Human Cancer Cell Line Preparation.

The HEK-293, MCF-7, and HeLa cells were used in human cancer cell line classification. Cell lines were cultured with growth media (Dulbecco’s modified Eagle’s medium [DMEM], 10% fetal bovine serum, 1% penicillin streptomycin) in a 10-cm Petri dish to 90% confluency before harvesting. After culturing, cell lines were harvested and resuspended to a concentration of ∼1 × 10^6^ cells/mL in 1× phosphate-buffered saline (PBS). The CFSE cell proliferation kit (Ex/Em 492/517 nm, Cat. 34554, Thermo Fisher) was added to the cell suspension at a working concentration of 20 μM. For the CellTrace yellow proliferation kit (Ex/Em 546/579 nm, Cat. 34567, Thermo Fisher), we prepared the CellTrace stock solution immediately prior to use by adding the appropriate volume of dimethyl sulfoxide (component B) to one vial of CellTrace reagent (component A) and then added the solution to the cell suspension at a working concentration of 5 μM. After incubating the cells at 37 °C for 30 min, fresh DMEM was used to quench the staining process, and the cells were washed with 1× PBS and fixed by a 4% paraformaldehyde solution. The fixed cells were washed and resuspended in 1× PBS before imaging.

### Human Breast Epithelial Cell Preparation.

The human breast epithelial cells (MCF-10A) were cultured with culture media (DMEM/F12 Ham’s mixture supplemented with 5% equine serum [Gemini Bio], 20 ng/mL epidermal growth factor [Sigma], 10 μg/mL insulin [Sigma], 0.5 mg/mL hydrocortisone [Sigma], 100 ng/mL cholera toxin [Sigma], 100 units/mL penicillin, and 100 μg/mL streptomycin) in a 15-cm Petri dish to 90% confluency before harvesting. After culturing, cells were harvested and resuspended to a concentration of ∼1 × 10^6^ cells/mL in 1× PBS. The cells were then fixed by a 4% paraformaldehyde solution. The fixed cells were washed and resuspended in 1× PBS before imaging.

### Stellate Cell Sample Preparation.

Frozen vials of SCs were first thawed in a 37 °C water bath and then transferred to 5 mL media (DMEM, 10% fetal bovine serum). Cells were then spun at 200 g for 5 min. The supernatant was removed, and the cells are resuspended in 1 mL of the medium. They were then transferred to 2 mL of Williams E Medium.

### Experimental Sample Preparation.

The cell sample was premixed with three nonfluorescent beads of different sizes, as follows: 10-µm beads which we represented as nucleobase A, 20-µm beads which we represented as nucleobase T, and 30-µm beads which we represented as nucleobase C. To keep the average number of cells between marker beads to be a relatively small number ($\bar{n}=2$) and minimize the chance of error, the ratio between cells and the total number of marker beads was 2:1.

## Supplementary Material

Supplementary File

## Data Availability

The data and Python implementation of unsupervised *k*-means clustering code are publicly available on GitHub (https://github.com/ZunmingZhang/A-High-Throughput-Technique-to-Map-Cell-Images-to-Cell-Positions-Using-3D-Imaging-Flow-Cytometer).
