# Supplementary material for: A high-throughput technique to map cell images to cell positions using a 3D imaging flow cytometer
Source: Proc Natl Acad Sci U S A. 2022 Feb 16;119(8):e2118068119. doi: 10.1073/pnas.2118068119 (PMC8872737; doi:10.1073/pnas.2118068119)
Supplement: Supplementary File [file pnas.2118068119.sapp.pdf]

PNAS
www.pnas.org

Supplementary Information for

A High Throughput Technique to Map Cell Images to Cell Positions Using 3D Imaging Flow Cytometer

Zunming Zhang[1], Rui Tang[1], Xinyu Chen[1], Lauren Waller[2], Alston Kau[3], Anthony A. Fung[2], Bien Gutierrez[3], Cheolhong An[1], Sung Hwan Cho[3], Lingyan Shi[2] and Yu-Hwa Lo[1,a)]

[1] Department of Electrical and Computer Engineering, University of California, San Diego, La Jolla, California 92093, USA; [2] Department of Bioengineering, University of California, San Diego, La Jolla, California 92093, USA; [3] NanoCellect Biomedical Inc., San Diego, California 92121, USA

[a)] Author to whom correspondence should be addressed.

Email: ylo@ucsd.edu

This PDF file includes:

Supplementary text
Figures S1 to S2

Supplementary Information Text

Cell Culturing and Sample Preparation Protocols

Human cancer cell line culturing

The human embryonic kidney 293 cells (HEK-293), the Michigan Cancer Foundation-7 cells (MCF-7), and the cervical cancer cells (HeLa) were used in human cancer cell line classification. Cell lines were all cultured with culture media (DMEM, 10% Fetal Bovine Serum, 1% Penicillin Streptomycin) in a 10 cm petri dish to 90% confluency before harvesting.

The human breast epithelial cells (MCF-10A) culturing

The human breast epithelial cells (MCF-10A) were cultured with culture media (DMEM /F12 Ham's Mixture supplemented with 5% Equine Serum (Gemini Bio), EGF 20 ng/ml(Sigma), insulin 10μg/ml (Sigma), hydrocortisone 0.5 mg/ml (Sigma), cholera toxin 100 ng/ml (Sigma), 100 units/ml penicillin and 100 μg/ml streptomycin) in a 15 cm petri dish to 90% confluency before harvesting. After culturing, cells were harvested and resuspended to a concentration of ~1x106 cells/mL in 1X PBS. The cells are then fixed by 4% paraformaldehyde solution. The fixed cells were washed and resuspended in 1X PBS before imaging.

Cell line CFSE staining and CellTrace Yellow staining

After culturing, Cell lines were harvested and resuspended to a concentration of ~1x106 cells/mL in 1X PBS. The CFSE Cell Proliferation Kit (Ex/Em 492/517nm, Cat. 34554, Thermo Fisher) was added to the cell suspension at a working concentration of 20μM. For the CellTrace Yellow Proliferation Kit (Ex/Em 546/579nm, Cat. 34567, Thermo Fisher), we prepared CellTrace stock solution immediately prior to use by adding the appropriate volume of DMSO (Component B) to one vial of CellTrace reagent (Component A) and then added the solution to the cell suspension at a working concentration of 5 μM. After incubating the cells at 37℃ for 30 minutes, fresh culture medium (DMEM) was used to quench the staining process, and the cells were washed with 1X PBS and fixed by 4% paraformaldehyde solution. The fixed cells were washed and resuspended in 1X PBS before imaging.

Tracking 3D cell images and their position experiment sample preparation

HeLa, MCF-7, and HEK-293 cells are used in this experiment. For the ground truth labeling HeLa and MCF-7 were fluorescently stained with the carboxyfluorescein succinimidyl ester (CFSE) Cell Proliferation Kit (Ex/Em 492/517 nm, Cat. 34554, Thermo Fisher) and the CellTrace Yellow Proliferation Kit (Ex/Em 546/579 nm, Cat. 34567, Thermo Fisher), respectively while leaving the HEK-293 to be unstained. After the staining and fixation, cells are mixed to a 1-1-1 ratio before the experiment.

Separating breast cancer cells from normal cells experiment sample preparation

MCF-7 and MCF-10A are used in this experiment. MCF-7 cells were fluorescently stained with the carboxyfluorescein succinimidyl ester (CFSE) Cell Proliferation Kit (Ex/Em 492/517 nm, Cat. 34554, Thermo Fisher), and the MCF-10A cells are not stained. After staining and fixation, MCF-7 and MCF-10A cells are mixed to a 1-1 ratio for the experiment.

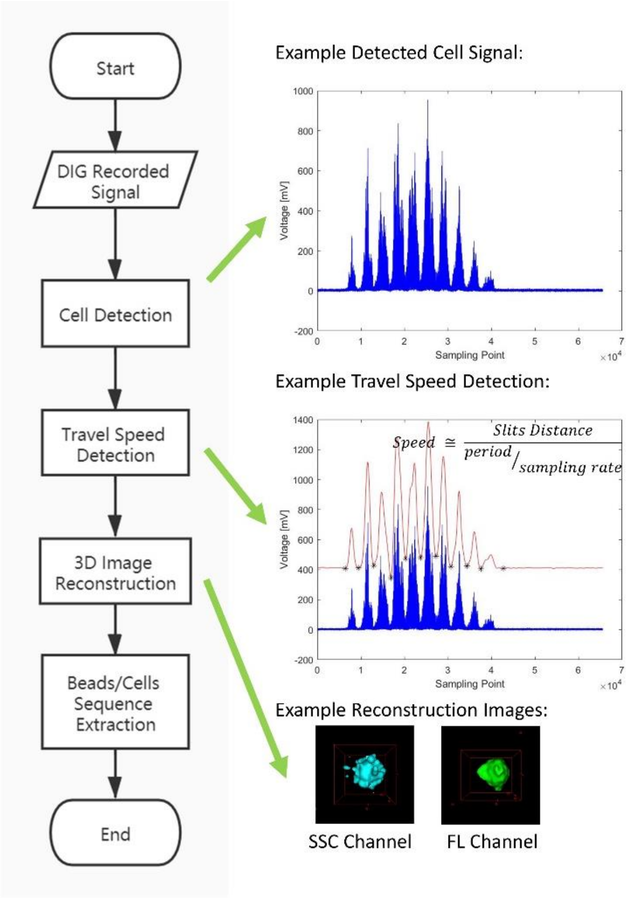

Fig. S1. Sequence extraction and image acquisition pipeline from 3D imaging flow cytometry (3D-IFC). Also shown are examples for detected cell signal waveform after applying the cell detection algorithm and cell speed detection. For cell speed detection, the design uses the following parameters: slits distance, 20um; sampling rate, 25M Samples/sec; period, the average number of sampling points between two adjacent troughs (represented as *).

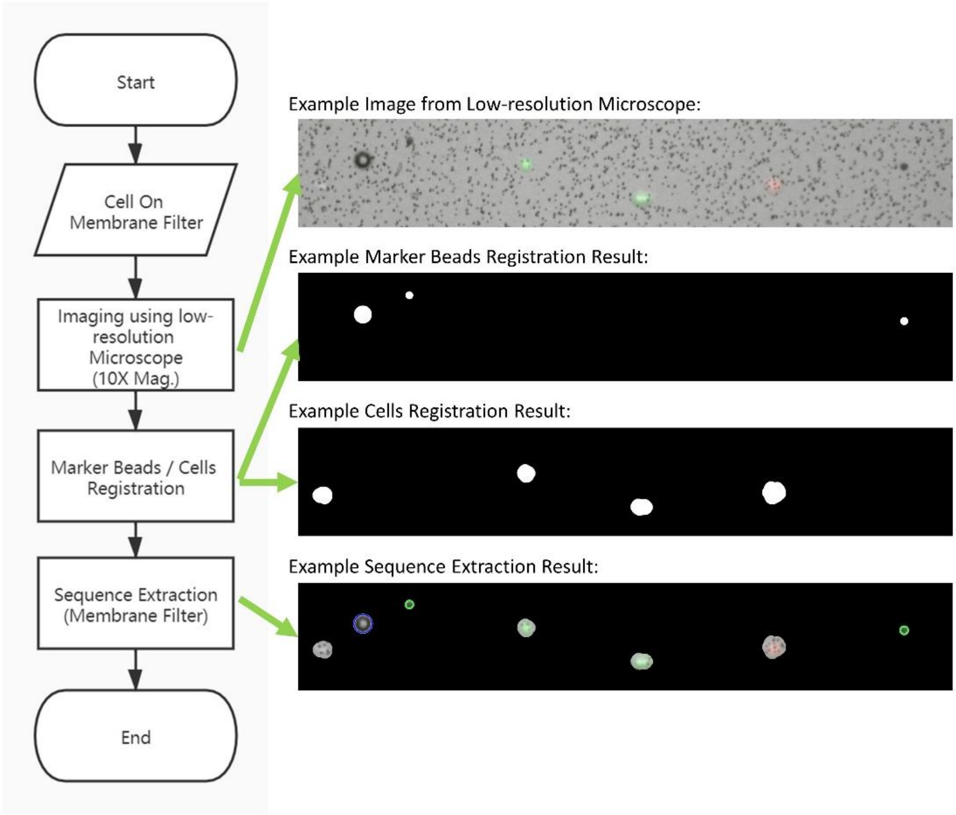

Fig. S2. Sequence extraction and marker bead and cell detection/ pipeline from cell placement platform (CPP). The coordinates of the beads/cells on the porous membrane are registered and the background interferences from the porous membrane are removed by the algorithm.
